# 2,6-Dichloro­pyridine-3,5-dicarbonitrile

**DOI:** 10.1107/S160053681003758X

**Published:** 2010-09-25

**Authors:** Adrian Woiczechowski-Pop, Richard A. Varga, Anamaria Terec, Ion Grosu

**Affiliations:** a Organic Chemistry Department, Faculty of Chemistry and Chemical Engineering, Babes Bolyai University, Arany Janos 11, 400028, Cluj Napoca, Romania; b Inorganic Chemistry Department, Faculty of Chemistry and Chemical Engineering, Babes Bolyai University, Arany Janos 11, 400028, Cluj Napoca, Romania

## Abstract

In the crystal, essentially planar (r.m.s. deviation = 0.003 Å) mol­ecules of the title compound, C_7_HCl_2_N_3_, form chains along the *b* axis by means of C—H⋯N inter­actions. These chains are further linked into layers parallel to the *ab* plane by C—Cl⋯N inter­actions.

## Related literature

For the structures of related pyridine derivatives, see: Boer *et al.* (1972 ▶); Clegg *et al.* (1997 ▶); Julia *et al.* (1983 ▶); Schlosser *et al.* (2006 ▶); Schmidt *et al.* (2005 ▶); Smith *et al.* (2008 ▶). For more information on the synthesis of 2,6-dichloro­pyridine-3,5-dicarbonitrile, see: Duindam *et al.* (1993 ▶). For compounds obtained from 2,6-dichloro­pyridine-3,5-dicarbonitrile, see: Katz *et al.* (2005 ▶); Vilarelle *et al.* (2004 ▶).
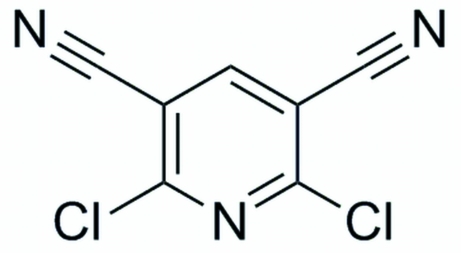


## Experimental

### 

#### Crystal data

C_7_HCl_2_N_3_
*M* *_r_* = 198.01
Orthorhombic, 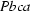

*a* = 6.8473 (9) Å
*b* = 12.1307 (15) Å
*c* = 19.430 (3) Å
*V* = 1613.9 (4) Å^3^
*Z* = 8
Mo *K*α radiation
μ = 0.74 mm^−1^
*T* = 297 K
0.41 × 0.39 × 0.39 mm

#### Data collection

Bruker SMART APEX CCD area-detector diffractometer
Absorption correction: multi-scan (*SADABS*; Bruker, 2000 ▶) *T*_min_ = 0.751, *T*_max_ = 0.761
10614 measured reflections
1420 independent reflections
1328 reflections with *I* > 2σ(*I*)
*R*_int_ = 0.049

#### Refinement

*R*[*F* ^2^ > 2σ(*F* ^2^)] = 0.067
*wR*(*F* ^2^) = 0.133
*S* = 1.29
1420 reflections
109 parameters
H-atom parameters constrained
Δρ_max_ = 0.25 e Å^−3^
Δρ_min_ = −0.33 e Å^−3^

### 

Data collection: *SMART* (Bruker, 2000 ▶); cell refinement: *SAINT-Plus* (Bruker, 2001 ▶); data reduction: *SAINT-Plus*; program(s) used to solve structure: *SHELXS97* (Sheldrick, 2008 ▶); program(s) used to refine structure: *SHELXL97* (Sheldrick, 2008 ▶); molecular graphics: *DIAMOND* (Brandenburg & Putz, 2006 ▶); software used to prepare material for publication: *publCIF* (Westrip, 2010 ▶).

## Supplementary Material

Crystal structure: contains datablocks I, New_Global_Publ_Block. DOI: 10.1107/S160053681003758X/ya2129sup1.cif

Structure factors: contains datablocks I. DOI: 10.1107/S160053681003758X/ya2129Isup2.hkl

Additional supplementary materials:  crystallographic information; 3D view; checkCIF report

## Figures and Tables

**Table 1:** Intermolecular interactions (Å, °)

*D*—*X*⋯*A*	*D*—*X*	*X*⋯*A*	*D*⋯*A*	*D*—*X*⋯*A*
C3—H3⋯N1^i^	0.93	2.54	3.412 (5)	157
C1—Cl1⋯N2^ii^	1.71 (1)	3.24 (1)	4.820 (5)	152 (1)
C5—Cl2⋯N3^iii^	1.72 (1)	3.28 (1)	4.851 (6)	151 (1)

Symmetry codes: (i) 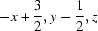; (ii) 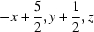; (iii) 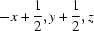.

**Fig. 1.**
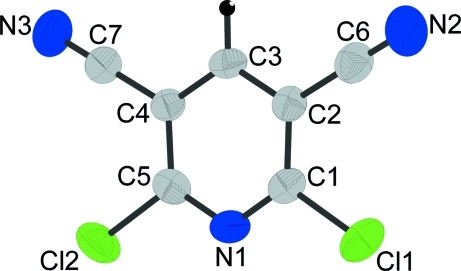
Molecular structure of the title compound; displacement ellipsoids are drawn at the 50% probability level, and the H3 atom is shown as a circle of arbitrary small radius.

**Fig. 2.**
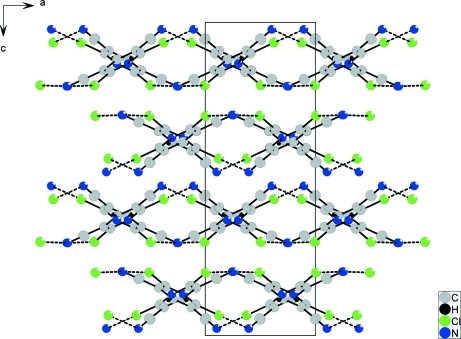
Packing diagram for the crystal of the title compound viewed down the b axis. The C—H···N and Cl···N interactions are shown as dashed lines.

## supplementary crystallographic information

### Comment

Some pyridine derivatives are known to be important intermediates in pharmaceutical and medicinal chemistry. They can be used for the synthesis of various compounds having antibacterial, antianaphilactic, antipyretic, antiallergic or anticancer properties (Vilarelle *et al.*, 2004). Lately, pyridine derivatives were used in the synthesis of cage molecules, supramolecular structures which are important as tools for the study of molecular encapsulation and host–guest interactions (Katz *et al.*, 2005).

Relatively few crystal structures of pyridines substituted in positions 2 and 6 with chlorine atoms were published (Boer *et al.*, 1972; Clegg *et al.*, 1997; Julia *et al.*, 1983; Schlosser *et al.*, 2006; Schmidt *et al.*, 2005; Smith *et al.*, 2008). The synthesis of the title compound has been reported some years ago (Duindam *et al.*, 1993), however its crystal structure has not yet been determined, even though electron withdrawing CN groups, causing considerable acidity of the H atom in position 4, as well as chlorine substituents at the pyridine atoms adjacent to the endocyclic *N* atom, may give rise to important non-conventional intermolecular interactions. Therefore, one may expect that structural study of the title compound may provide some non-trivial information.

Molecule of the title compound (Fig. 1) is essentially planar; the N2 and N3 atoms deviate from the pyridine plane by 0.060 (4) Å and 0.026 (4) Å respectively.

The molecules are linked by means of C3—H3···N1^i^ ineractions (Table 1) into infinite chains running along the *b* axis. The pyridine rings in the adjacent molecules of these chains are not coplanar, but rather form substantial dihedral angle with one another [56.5 (1)°]. The chains are further linked *via* C1—Cl1···N2^ii^ and C5—Cl2···N3^iii^ interactions into layers parallel to the *ab*-plane (Table 1; Fig. 2).

### Experimental

A mixture of malononitrile (5 g, 75.69 mmol, 2 equiv.), triethyl orthoformate (5.61 g, 37.84 mmol, 1 equiv.) and pyridine (2.99 g, 37.84 mmol, 1 equiv.) was allowed to reflux for 20 minutes, then concentrated HCl was added at 80°C. The mixture was cooled to room temperature and water (20 ml) was added. The formed precipitate was collected by filtration, washed successively with water, ethanol and diethylether to afford the intermediate 2-amino-6-chloropyridine-3,5-dicarbonitrile (9.74 g, 96%). To a solution of 2-amino-6-chloropyridine-3,5-dicarbonitrile (3 g, 16.8 mmol, 1 equiv.) and CuCl_2_ (3.39 g, 25.2 mmol, 1.5 equiv.) in dry CH_3_CN (150 ml), isopentyl nitrite was added (2.95 g, 25.2 mmol, 1.5 equiv.). The mixture was heated at 65°C for 5 h. The solution was acidified (HCl, 2 N) to pH=3, extracted with CH_2_Cl_2_ (3×50 ml) and dried with Na_2_SO_4_. The solvent was removed under reduced pressure and the crude product was purified by flash chromatography using CH_2_Cl_2_ as eluent to yield 2,6-dichloropyridine-3,5-dicarbonitrile as a colourless solid (2.97 g, 89%). The crystals were obtained by slow evaporation of the solvent from solution of the title compound in dichloromethane.

### Refinement

The H3 atom was placed in calculated position (C—H = 0.93 Å) and treated in the subsequent refinement using the riding model approximation with *U*_iso_= 1.2*U*_eq_(C).

### Crystal data

**Table:**

C_7_HCl_2_N_3_	*F*(000) = 784
*M**_r_* = 198.01	*D*_x_ = 1.630 Mg m^−^^3^
Orthorhombic, *P**b**c**a*	Mo *K*α radiation, λ = 0.71073 Å
Hall symbol: -P 2ac 2ab	Cell parameters from 4078 reflections
*a* = 6.8473 (9) Å	θ = 3.1–26.1°
*b* = 12.1307 (15) Å	µ = 0.74 mm^−^^1^
*c* = 19.430 (3) Å	*T* = 297 K
*V* = 1613.9 (4) Å^3^	Block, colourless
*Z* = 8	0.41 × 0.39 × 0.39 mm

### Data collection

**Table:**

Bruker SMART APEX CCD area-detector diffractometer	1420 independent reflections
Radiation source: fine-focus sealed tube	1328 reflections with *I* > 2σ(*I*)
graphite	*R*_int_ = 0.049
phi and ω scans	θ_max_ = 25.0°, θ_min_ = 2.1°
Absorption correction: multi-scan (*SADABS*; Bruker, 2000)	*h* = −8→8
*T*_min_ = 0.751, *T*_max_ = 0.761	*k* = −14→14
10614 measured reflections	*l* = −23→23

### Refinement

**Table:**

Refinement on *F*^2^	Primary atom site location: structure-invariant direct methods
Least-squares matrix: full	Secondary atom site location: difference Fourier map
*R*[*F*^2^ > 2σ(*F*^2^)] = 0.067	Hydrogen site location: inferred from neighbouring sites
*wR*(*F*^2^) = 0.133	H-atom parameters constrained
*S* = 1.29	*w* = 1/[σ^2^(*F*_o_^2^) + (0.0317*P*)^2^ + 2.8365*P*] where *P* = (*F*_o_^2^ + 2*F*_c_^2^)/3
1420 reflections	(Δ/σ)_max_ < 0.001
109 parameters	Δρ_max_ = 0.25 e Å^−^^3^
0 restraints	Δρ_min_ = −0.33 e Å^−^^3^

### Special details

**Table:**

Geometry. All e.s.d.'s (except the e.s.d. in the dihedral angle between two l.s. planes) are estimated using the full covariance matrix. The cell e.s.d.'s are taken into account individually in the estimation of e.s.d.'s in distances, angles and torsion angles; correlations between e.s.d.'s in cell parameters are only used when they are defined by crystal symmetry. An approximate (isotropic) treatment of cell e.s.d.'s is used for estimating e.s.d.'s involving l.s. planes.
Refinement. Refinement of *F*^2^ against ALL reflections. The weighted *R*-factor *wR* and goodness of fit *S* are based on *F*^2^, conventional *R*-factors *R* are based on *F*, with *F* set to zero for negative *F*^2^. The threshold expression of *F*^2^ > σ(*F*^2^) is used only for calculating *R*-factors(gt) *etc*. and is not relevant to the choice of reflections for refinement. *R*-factors based on *F*^2^ are statistically about twice as large as those based on *F*, and *R*- factors based on ALL data will be even larger.

### Fractional atomic coordinates and isotropic or equivalent isotropic displacement parameters (Å^2^)

**Table:**

	*x*	*y*	*z*	*U*_iso_*/*U*_eq_	
C1	0.9151 (5)	0.3589 (3)	0.4007 (2)	0.0375 (9)	
C2	0.8734 (5)	0.2479 (3)	0.40946 (19)	0.0364 (9)	
C3	0.7015 (5)	0.2084 (3)	0.38179 (18)	0.0368 (9)	
H3	0.6687	0.1343	0.3861	0.044*	
C4	0.5791 (5)	0.2801 (3)	0.34764 (18)	0.0332 (8)	
C5	0.6383 (6)	0.3884 (3)	0.34168 (19)	0.0390 (9)	
C6	1.0034 (6)	0.1770 (4)	0.4467 (2)	0.0474 (10)	
C7	0.3992 (6)	0.2425 (3)	0.3184 (2)	0.0438 (10)	
Cl1	1.12351 (16)	0.41416 (10)	0.43512 (6)	0.0578 (4)	
Cl2	0.49486 (18)	0.48205 (10)	0.29881 (6)	0.0604 (4)	
N1	0.8014 (5)	0.4280 (3)	0.36795 (16)	0.0404 (8)	
N2	1.1049 (6)	0.1222 (3)	0.4775 (2)	0.0683 (12)	
N3	0.2550 (6)	0.2128 (4)	0.29590 (19)	0.0630 (11)	

### Atomic displacement parameters (Å^2^)

**Table:**

	*U*^11^	*U*^22^	*U*^33^	*U*^12^	*U*^13^	*U*^23^
C1	0.033 (2)	0.040 (2)	0.039 (2)	−0.0043 (18)	0.0058 (17)	−0.0067 (17)
C2	0.035 (2)	0.0368 (19)	0.0372 (19)	0.0031 (18)	−0.0002 (16)	−0.0039 (17)
C3	0.042 (2)	0.0271 (18)	0.041 (2)	−0.0021 (17)	0.0037 (18)	−0.0046 (16)
C4	0.0343 (19)	0.0314 (19)	0.0339 (19)	0.0003 (16)	0.0020 (16)	−0.0033 (15)
C5	0.041 (2)	0.037 (2)	0.039 (2)	0.0048 (18)	0.0058 (18)	0.0025 (17)
C6	0.048 (2)	0.047 (2)	0.048 (2)	0.003 (2)	−0.003 (2)	−0.009 (2)
C7	0.048 (2)	0.041 (2)	0.042 (2)	−0.001 (2)	−0.003 (2)	−0.0012 (18)
Cl1	0.0412 (6)	0.0622 (7)	0.0699 (8)	−0.0128 (5)	−0.0045 (5)	−0.0138 (6)
Cl2	0.0611 (7)	0.0487 (6)	0.0714 (8)	0.0081 (6)	−0.0105 (6)	0.0191 (5)
N1	0.0417 (19)	0.0334 (17)	0.0461 (19)	−0.0030 (15)	0.0061 (16)	−0.0011 (15)
N2	0.067 (3)	0.064 (3)	0.074 (3)	0.025 (2)	−0.020 (2)	−0.007 (2)
N3	0.054 (2)	0.075 (3)	0.060 (2)	−0.014 (2)	−0.013 (2)	0.002 (2)

### Geometric parameters (Å, °)

**Table:**

C1—N1	1.309 (5)	C4—C5	1.380 (5)
C1—C2	1.387 (5)	C4—C7	1.430 (5)
C1—Cl1	1.713 (4)	C5—N1	1.319 (5)
C2—C3	1.380 (5)	C5—Cl2	1.717 (4)
C2—C6	1.434 (6)	C6—N2	1.133 (5)
C3—C4	1.378 (5)	C7—N3	1.139 (5)
C3—H3	0.9300		
			
N1—C1—C2	124.0 (4)	C3—C4—C5	117.6 (3)
N1—C1—Cl1	115.8 (3)	C3—C4—C7	120.9 (3)
C2—C1—Cl1	120.2 (3)	C5—C4—C7	121.6 (3)
C3—C2—C1	117.7 (3)	N1—C5—C4	124.3 (4)
C3—C2—C6	121.2 (4)	N1—C5—Cl2	115.5 (3)
C1—C2—C6	121.1 (4)	C4—C5—Cl2	120.2 (3)
C4—C3—C2	119.1 (3)	N2—C6—C2	178.4 (5)
C4—C3—H3	120.4	N3—C7—C4	179.2 (5)
C2—C3—H3	120.4	C1—N1—C5	117.3 (3)
			
N1—C1—C2—C3	0.1 (6)	C3—C4—C5—N1	−1.8 (6)
Cl1—C1—C2—C3	178.7 (3)	C7—C4—C5—N1	179.4 (4)
N1—C1—C2—C6	−179.2 (4)	C3—C4—C5—Cl2	179.1 (3)
Cl1—C1—C2—C6	−0.6 (5)	C7—C4—C5—Cl2	0.3 (5)
C1—C2—C3—C4	−0.6 (5)	C2—C1—N1—C5	−0.3 (6)
C6—C2—C3—C4	178.7 (4)	Cl1—C1—N1—C5	−179.0 (3)
C2—C3—C4—C5	1.4 (5)	C4—C5—N1—C1	1.2 (6)
C2—C3—C4—C7	−179.8 (3)	Cl2—C5—N1—C1	−179.6 (3)

### Hydrogen-bond geometry (Å, °)

**Table:**

*D*—H···*A*	*D*—H	H···*A*	*D*···*A*	*D*—H···*A*
C3—H3···N1^i^	0.93	2.54	3.412 (5)	157
C1—Cl1···N2^ii^	1.713 (4)	3.241 (4)	4.820 (5)	151.9 (2)
C5—Cl2···N3^iii^	1.717 (4)	3.281 (5)	4.851 (6)	150.6 (2)

Symmetry codes: (i) −*x*+3/2, *y*−1/2, *z*; (ii) −*x*+5/2, *y*+1/2, *z*; (iii) −*x*+1/2, *y*+1/2, *z*.
